# 972. Antimicrobial Stewardship Educational Needs of Residents in an Internal Medicine Program

**DOI:** 10.1093/ofid/ofab466.1167

**Published:** 2021-12-04

**Authors:** Jessica Kennedy, Pranisha Gautam-Goyal, Robin V Koshy, Thien-Ly Doan, Neha Paralkar, Karen Friedman

**Affiliations:** 1 Zucker School of Medicine at Northwell, Manhasset, New York; 2 Long Island Jewish Medical Center, New Hyde Park, New York

## Abstract

**Background:**

Antibiotic stewardship continues to be health concern that physicians often acknowledge, but whose real-life practices do not reflect that awareness. There is a wide range of opinions on the efficacy of the type of modality that is most effective to teach stewardship. Our project addresses resident needs specifically, with coverage in four topics—proper antibiotic dosing, IV to PO transitioning, duplicate coverage, and antibiotic time outs.

**Methods:**

Categorical Internal Medicine residents in PGY 1-3 were sent an optional 48-question Likert survey querying needs in the above four topics.

**Results:**

General Demographics. Resident response was 35%, with equal representation from all PGY years. Over half reported no ID or stewardship elective exposure and 74% agreed they could benefit from further education on stewardship (Figure 1). Proper Dosing Educational Needs. Of residents, 68% reported feeling confident about where to find information on dosing antibiotics for a given condition/organism (Figure 2a), but only 37% were comfortable with establishing an initial dose. When a range was suggested, 55% of respondents admitted to at least “sometimes, often, or always” choosing the highest suggested dose by default. IV to PO transition. Residents preferred (76%) and used (89%) IV antibiotics by default in an inpatient setting. Nearly 45% of respondents reported “sometimes or rarely” feeling comfortable in making an IV to PO transition, and 40% “often or always” avoid PO transition until discharge (Figure 2b). Duplicate Coverage. Over 70% of residents reported they “sometimes, rarely, or never” felt confident in stopping double coverage themselves when started by the primary team (Figure 3a). Antibiotic Time Out. Only 17% of respondents had heard of an antibiotic timeout, and only 8% have ever used one (Fig.3b); 80% of residents had no structured way to review usage and 53% reported “sometimes or often” forgetting about assessing for de-escalation daily.

Figure 1. Resident Demographics

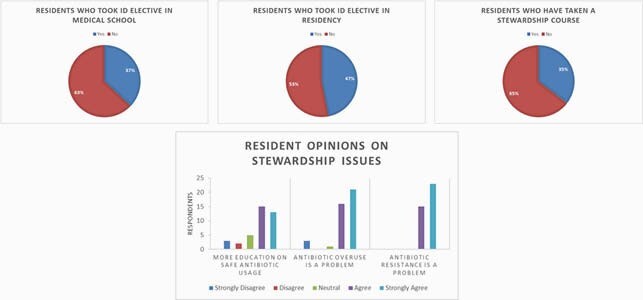

Our anonymous, optional survey attracted a 35% response rate from the categorical residents at our suburban program spread over two tertiary hospitals with >1200 beds total. Most had not received prior training in infectious disease or stewardship, yet most recognized antibiotic overuse and resistance as a major, ongoing problem.

Figure 2. Resident responses on proper dosing and IV to PO questions.

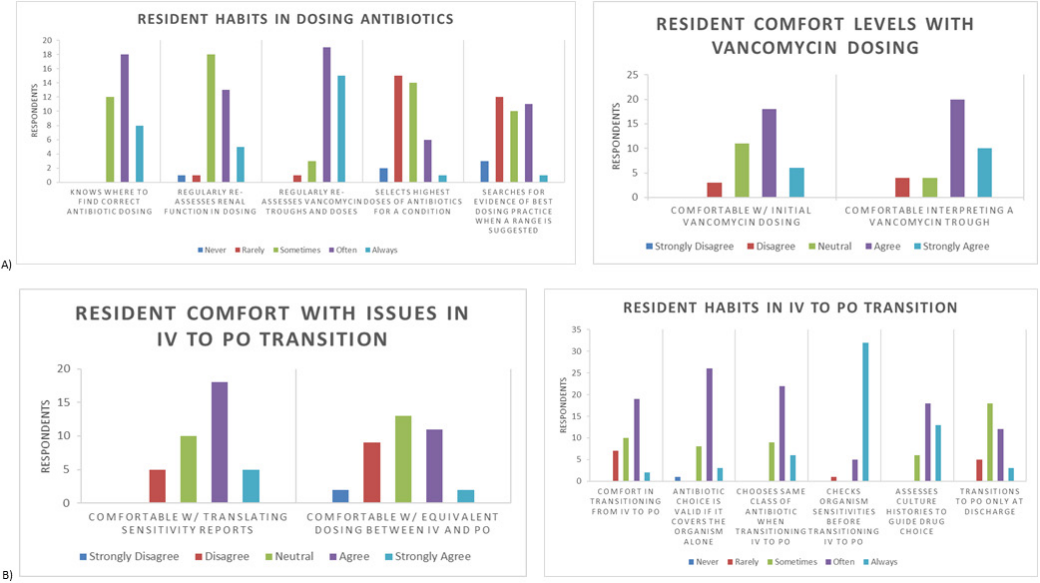

(A) Residents appear most uncomfortable with initial antibiotic dosing and seeking additional sources for best dosage when commonly used sources suggest a range of possible doses. (B) Majority of residents preferred and used IV antibiotics, and commonly transitioned to PO only at patient discharge. Some residents reported discomfort with establishing equivalent IV to PO transition dosages.

Figure 3. Resident responses to questions regarding duplication of therapy and antibiotic time outs.

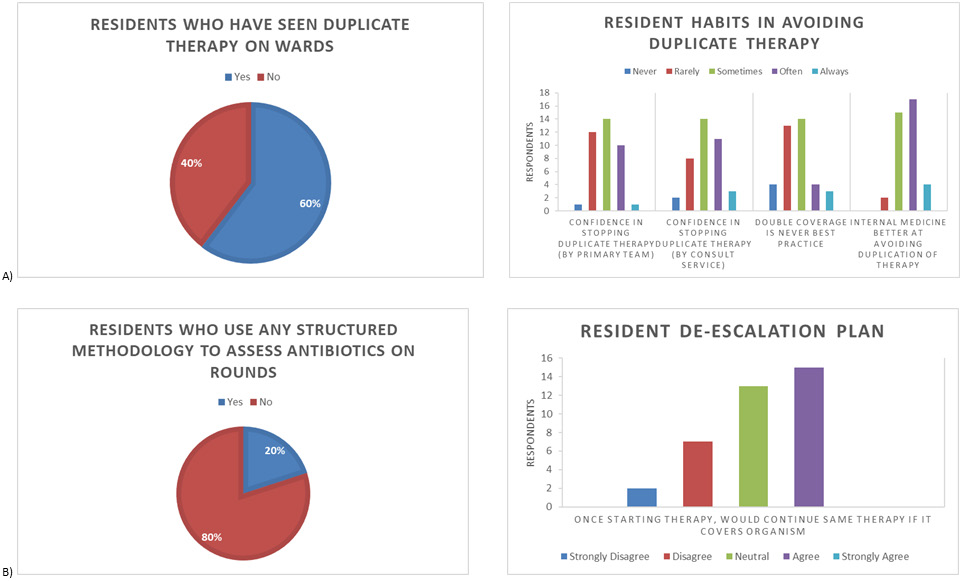

(A) Though many could and had recognized duplication of therapy on the wards, several participants reported at least some discomfort in independently stopping double coverage. (B) Most residents had not heard of or utilized an antibiotic time-out or any other structured method to re-assess their antibiotic use on daily rounds. As such, 41% of respondents admitted they would likely just continue initial, broad-spectrum therapy.

**Conclusion:**

Our analysis aimed to establish resident educational needs in four major topics in stewardship. Gaps in knowledge include timing transition from IV to PO, initial antibiotic dosing, stopping double-coverage, and lack of awareness of timeouts. This needs assessment will be used to build an antibiotic stewardship curriculum for IM residents.

**Disclosures:**

**All Authors**: No reported disclosures

